# Acute post-procedural inducibility is a poor predictor of clinical outcomes in high-risk patients (PAINESD > 17) undergoing scar-related ventricular tachycardia ablation

**DOI:** 10.1093/europace/euae185

**Published:** 2024-07-20

**Authors:** Joseph Sipko, Bryan Baranowski, Mandeep Bhargava, Thomas D Callahan, Thomas J Dresing, Koji Higuchi, Ayman A Hussein, Mohamed Kanj, Justin Lee, David O Martin, Shady Nakhla, John J Rickard, Walid I Saliba, Tyler Taigen, Oussama M Wazni, Pasquale Santangeli, Jakub Sroubek

**Affiliations:** Section of Cardiac Pacing and Electrophysiology, Cleveland Clinic Foundation, Cleveland, OH, USA; Section of Cardiac Pacing and Electrophysiology, Cleveland Clinic Foundation, Cleveland, OH, USA; Section of Cardiac Pacing and Electrophysiology, Cleveland Clinic Foundation, Cleveland, OH, USA; Section of Cardiac Pacing and Electrophysiology, Cleveland Clinic Foundation, Cleveland, OH, USA; Section of Cardiac Pacing and Electrophysiology, Cleveland Clinic Foundation, Cleveland, OH, USA; Section of Cardiac Pacing and Electrophysiology, Cleveland Clinic Foundation, Cleveland, OH, USA; Section of Cardiac Pacing and Electrophysiology, Cleveland Clinic Foundation, Cleveland, OH, USA; Section of Cardiac Pacing and Electrophysiology, Cleveland Clinic Foundation, Cleveland, OH, USA; Section of Cardiac Pacing and Electrophysiology, Cleveland Clinic Foundation, Cleveland, OH, USA; Section of Cardiac Pacing and Electrophysiology, Cleveland Clinic Foundation, Cleveland, OH, USA; Section of Cardiac Pacing and Electrophysiology, Cleveland Clinic Foundation, Cleveland, OH, USA; Section of Cardiac Pacing and Electrophysiology, Cleveland Clinic Foundation, Cleveland, OH, USA; Section of Cardiac Pacing and Electrophysiology, Cleveland Clinic Foundation, Cleveland, OH, USA; Section of Cardiac Pacing and Electrophysiology, Cleveland Clinic Foundation, Cleveland, OH, USA; Section of Cardiac Pacing and Electrophysiology, Cleveland Clinic Foundation, Cleveland, OH, USA; Section of Cardiac Pacing and Electrophysiology, Cleveland Clinic Foundation, Cleveland, OH, USA; Section of Cardiac Pacing and Electrophysiology, Cleveland Clinic Foundation, Cleveland, OH, USA

**Keywords:** Ventricular tachycardia, Ablation, Inducibility, Procedural endpoint

## Abstract

**Aims:**

Ventricular tachycardia (VT) non-inducibility in response to programmed ventricular stimulation (PVS) is a widely used procedural endpoint for VT ablation despite inconclusive evidence with respect to clinical outcomes in high-risk patients. The aim is to determine the utility of acute post-ablation VT inducibility as a predictor of VT recurrence, mortality, or mortality equivalent in high-risk patients.

**Methods and results:**

We conducted a retrospective analysis of high-risk patients (defined as PAINESD > 17) who underwent scar-related VT ablation at our institution between July 2010 and July 2022. Patients’ response to PVS (post-procedure) was categorized into three groups: Group A, no clinical VT or VT with cycle length > 240 ms inducible; Group B, only non-clinical VT with cycle length > 240 ms induced; and Group C, all other outcomes (including cases where no PVS was performed). The combined primary endpoint included death, durable left ventricular assist device placement, and cardiac transplant (Cox analysis). Ventricular tachycardia recurrence was considered a secondary endpoint (competing risk analysis). Of the 1677 VT ablation cases, 123 cases met the inclusion criteria for analysis. During a 19-month median follow-up time (interquartile range 4–43 months), 82 (66.7%) patients experienced the composite primary endpoint. There was no difference between Groups A and C with respect to the primary [hazard ratio (HR) = 1.21 (0.94–1.57), *P* = 0.145] or secondary [HR = 1.18 (0.91–1.54), *P* = 0.210] outcomes. These findings persisted after multivariate adjustments. The size of Group B (*n* = 13) did not permit meaningful statistical analysis.

**Conclusion:**

The results of post-ablation PVS do not significantly correlate with long-term outcomes in high-risk (PAINESD > 17) VT ablation patients.

## Graphical Abstract

**Graphical Abstract:**
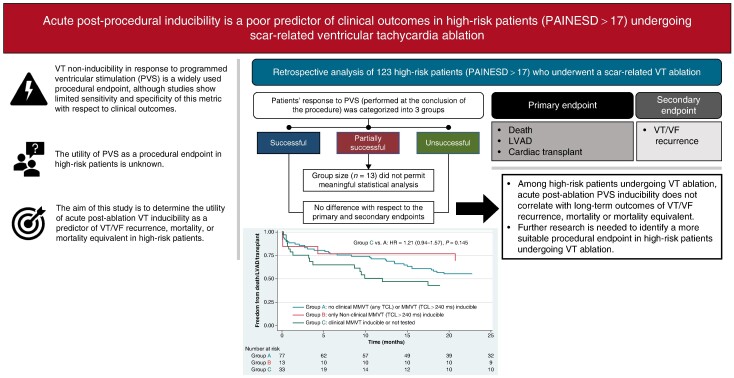

**What’s new?**

- Among high-risk patients (PAINESD > 17) undergoing ventricular tachycardia (VT) ablation, acute post-ablation programmed ventricular stimulation inducibility does not correlate with long-term outcomes of VT recurrence, mortality, or mortality equivalent.
- Further research is needed to identify a more suitable procedural endpoint in high-risk patients undergoing VT ablation.

## Introduction

Radiofrequency (RF) catheter ablation is a mainstay therapy for patients with scar-related ventricular tachycardia (VT), especially when refractory to antiarrhythmic drug therapy.^1,2^ Programmed ventricular stimulation (PVS) is a VT induction technique that enjoyed wide adoption since the 1970s and serves an integral part of most ablation procedures.^3,4^ In particular, VT non-inducibility in response to PVS is a widely used procedural endpoint for VT ablation,^5,6^ although there is inconclusive evidence to support PVS as a reliable risk stratifier for clinical outcomes.^7–13^ Furthermore, the utility of PVS as a procedural endpoint in high-risk patients, with multiple advanced comorbidities, is unknown. This analysis sought to determine the utility of acute post-ablation VT inducibility as a predictor of VT recurrence, mortality, or mortality equivalent in high-risk patients.

## Methods

### Study population

**Figure 1:**
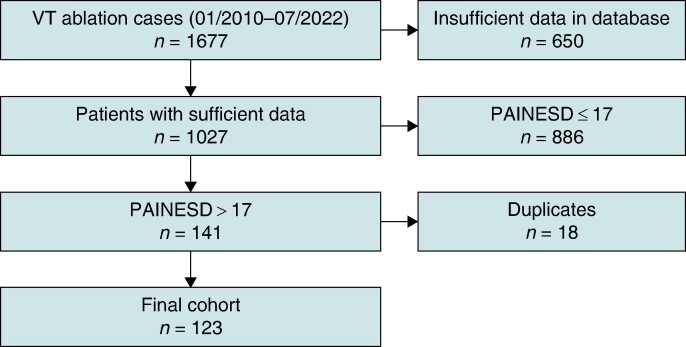
Study flowchart. VT, ventricular tachycardia.

We conducted retrospective analysis of high-risk patients, aged 18 years and above from January 2010 to July 2022, who underwent catheter ablation of VT at the Cleveland Clinic (CCF). The study was approved by the CCF Institutional Review Board. Patients were identified using the institution’s electrophysiology (EP) procedure database. High-risk patients were defined as PAINESD score > 17.^14^ Patients with PAINESD score of 17 or less, or cases with insufficient clinical data available for review, were excluded (*Figure 1*). Patients were included if they underwent VT ablation, and it was their first ablation at CCF (whether or not prior ablations were performed at another institution). Repeat VT ablations at CCF were excluded from analysis (to avoid data clustering).

Data were collected by accessing each patient’s medical record, including ablation procedure notes, clinic/hospital progress notes, and implantable cardioverter-defibrillator (ICD) interrogation reports. Data pertaining to PVS methods and outcomes were obtained by directly reviewing recording system tracings (CardioLab, GE HealthCare, Chicago, IL) whenever available. Primary inducibility data were reviewed by two independent investigators (Drs Sipko and Sroubek), and a consensus was reached in cases of discordant adjudication.

### Mapping and ablation strategy

All VT ablation procedures were performed according to the CCF practice standards as well as individual operators’ preferences tailored to each patient’s specific clinical needs. Procedures were performed under conscious sedation or general anaesthesia. Antiarrhythmic drugs were not routinely discontinued before the procedure given the high-risk nature of the studied population where VT storm was frequently the presenting condition prompting VT ablation. Anticoagulation was held for at least 24 h prior to the procedure, whenever clinically feasible. Mechanical circulatory support was used when clinically indicated. Femoral vascular access was obtained under ultrasound guidance. Epicardial access, when needed, was obtained using the modified Sosa technique.^15^ Catheters were advanced into the left ventricle (LV) via a retrograde aortic approach or via a trans-septal approach, per operator’s preference. Left ventricular mapping was done using a variety of mapping systems and compatible multipolar or ablation catheters: CARTO 3 using the PentaRay and/or DecaNav and/or ThermoCool ST/ST-SF catheters (Biosense Webster, Irvine, CA) and EnSite Precision using the Advisor HD Grid and/or TactiCath catheters (Abbott, Chicago, IL). When possible, operators were encouraged to determine baseline VT inducibility using PVS. Haemodynamically stable VT circuits were subjected to activation mapping and entrainment mapping, whenever possible. Substrate-based mapping techniques were also employed according to each operator’s choice (in general, cluster lesions were delivered targeting sites identified by pace mapping, as well as abnormal electrograms).

Radiofrequency ablation was performed in all cases using an externally irrigated ablation catheter [ThermoCool ST/ST SF (Biosense Webster, Irvine, CA) or TactiCath (Abbott, Chicago, IL)]; RF was typically delivered over 30–120 s per application, using 30–50 W output. Radiofrequency targets included putative VT critical sites as determined by VT activation mapping, VT entrainment mapping, and/or substrate mapping.

Post-ablation, repeat PVS was performed in stable patients; if clinically relevant sustained VT [as judged by the primary operator based on 12-lead electrocardiogram (ECG) morphology and tachycardia cycle length] was induced, additional RF delivery was performed until no further VT with tachycardia cycle length of ≤240 ms was inducible.

### Programmed ventricular stimulation

Programmed ventricular stimulation was repeated at the conclusion of each procedure unless the primary operator judged this manoeuver to be clinically inappropriate (e.g. if patient was haemodynamically unstable or if PVS was felt to be clinically futile due to inability to eliminate the clinical VT).

Programmed ventricular stimulation was performed from one to two pacing sites [e.g. right ventricular (RV) apex and lateral LV], using 8-beat drives delivered with at least two drive cycle lengths (e.g. 600 and 400 ms) and up to three extra-stimuli down to the ventricular effective refractory period or coupling intervals of 200 ms (whichever was higher). Programmed ventricular stimulation was stopped whenever any sustained ventricular arrhythmia was induced, including organized VT as well as ventricular fibrillation (VF).

For the purposes of this investigation, patients were categorized based on the outcome of PVS as follows:

- Group A: ‘successful ablation’ defined as inability to induce clinical VT and inability to induce non-clinical VT with cycle length > 240 ms (or tachycardia rate of 250 bpm or less).
- Group B: ‘partially successful ablation’ defined as inability to induce clinical VT but non-clinical VT with cycle length > 240 ms remained inducible.
- Group C: ‘unsuccessful ablation’ if clinical VT was inducible or patient was not tested for inducibility.

Patients without an inducible VT at the onset of the procedure who underwent a substrate-based ablation approach were assigned into Group A. The distinction between ‘clinical’ and ‘non-clinical’ was left up to the judgement of the primary operator. In general, a VT was considered to be ‘clinical’ if its surface 12-lead ECG morphology matched a documented spontaneous arrhythmia, if its surface 12-lead ECG morphology suggested a similar origin as a documented spontaneous arrhythmia (e.g. the same substrate but different exit), and/or if its cycle length and electrogram morphology and relative timing were similar to the cycle length and electrogram morphology and relative timing of a spontaneous sustained VT documented with ICD interrogation.

### Study outcomes and follow-up

Short-term outcomes refer to a 30-day follow-up, while long-term outcomes refer to the period leading up to the most recent visit available in our electronic medical record.

The primary endpoints were (i) the combined endpoint of death, left ventricular assist device (LVAD), or cardiac transplant and (ii) VT/VF recurrence defined as appropriate ICD therapies or VT/VF requiring external cardioversion/defibrillation. Recurrence of VT/VF did not count towards the primary endpoint. Both outcomes were stratified by procedural success category based on post-ablation inducibility.

### Statistical analysis

Patient characteristics were represented with descriptive statistics. Continuous variables were described using mean [standard deviation (SD)] or median [interquartile range (IQR)]. Categorical variables were described as frequencies or proportions. Characteristics were compared between study groups using Student’s *t*-test, non-parametric testing (Wilcoxon test), or Fisher’s exact test, as appropriate. The univariate effect of VT inducibility on the primary endpoints was evaluated with event-free survival analysis using the Cox model (for the composite primary endpoint) and Fine–Gray competing risk model (for VT/VF recurrence as the outcome of interest; mortality equivalent was used as the competing event) for long-term outcomes reported at hazard ratios (HR) and 95% confidence intervals (CI). Logistic regression was used to assess the short-term (30-day) outcomes, reported as odds ratios (OR) and 95% CI. Exploratory secondary survival analysis was performed to assess 30-day VT/VF recurrence as a predictor of the composite outcome of death, LVAD, or cardiac transplant, using the Cox model. Multivariate analysis, adjusted for creatinine clearance, pericardial access, and mechanical support, was also used to assess event-free survival time with respect to the primary endpoints using the same models as detailed above. Statistical significance was assigned to *P* < 0.05.

## Results

### Patients

Of the 1677 VT/PVC ablation procedures performed from January 2010 to July 2022, 123 cases satisfied the previously defined inclusion criteria of being the first VT ablation at our institution in a high-risk patient (PAINESD score > 17; *Figure 1*).

Patients were stratified into three groups based on their acute post-procedural VT inducibility in response to PVS: Group A (successful ablation, *n* = 77), Group B (partially successful ablation, *n* = 13), and Group C (unsuccessful ablation, *n* = 33).

The study population consisted of 114 (92.7%) patients with ischaemic cardiomyopathy (ICM) and 97 (78.9%) patients with New York Heart Association (NYHA) functional Classes III and IV, with a median left ventricular ejection fraction (LVEF) of 25% (IQR 20–31%). Most patients presented in VT storm [*n* = 103 (83.7%)] shortly before the ablation procedure. There were no statistically significant differences in the baseline clinical characteristics of patients in Groups A and C (*Table 1*; Group B contained only 13 patients and was consequently excluded from statistical analyses).

**Table 1:** Baseline patient characteristics

Characteristic	Group A	Group B	Group C	All	*P*-value^a^
Number of patients, *n* (%)	77 (62.6)	13 (10.6)	33 (26.8)	123 (100)	—
Age in years, mean ± SD	69.4 ± 7.1	70.8 ± 6.5	70.7 ± 6.9	69.9 ± 6.9	0.393
Male sex, *n* (%)	73 (94.8)	12 (92.3)	29 (87.9)	114 (96.7)	0.238
Hypertension, *n* (%)	72 (93.5)	12 (92.3)	32 (97.0)	116 (94.3)	0.666
Diabetes, *n* (%)	37 (48.1)	4 (30.8)	12 (36.4)	53 (43.1)	0.299
Creatinine clearance in mL/min, mean ± SD	68.1 ± 35.1	55.6 ± 29.8	57.6 ± 34.1	63.9 ± 34.5	0.150
ESRD, *n* (%)	2 (2.6)	1 (7.7)	2 (6.1)	5 (4.1)	0.582
Pulmonary disease, *n* (%)	43 (55.8)	9 (69.2)	18 (54.6)	70 (56.9)	1.000
CVA/TIA, *n* (%)	9 (11.7)	1 (7.7)	4 (12.1)	14 (11.4)	1.000
Use of amiodarone before ablation, *n* (%)	61 (79.2)	12 (92.3)	26 (78.8)	99 (80.5)	1.000
Use of therapeutic anticoagulation, *n* (%)	38 (49.4)	7 (53.9)	13 (39.4)	58 (47.2)	0.406
Ischaemic cardiomyopathy, *n* (%)	71 (92.2)	12 (92.3)	31 (93.9)	114 (92.7)	1.000
NYHA functional Classes III and IV, *n* (%)	61 (79.2)	9 (69.2)	27 (81.8)	97 (78.9)	1.000
LVEF in %, median (IQR)	24 (20–32)	30 (20–40)	25 (20–30)	25 (20–31)	0.951
Prior cardiac surgery, *n* (%)	39 (50.7)	11 (84.6)	19 (57.6)	69 (56.1)	0.538
History of prior VT ablation					
At least one, *n* (%)	25 (32.5)	5 (38.5)	7 (21.2)	37 (43.0)	0.262
At least two, *n* (%)	9 (11.7)	3 (23.1)	0 (0.0)	12 (9.8)	0.055
VT storm on presentation, *n* (%)	65 (84.4)	12 (92.3)	26 (78.8)	103 (83.7)	0.583

CVA, cerebrovascular accident; IQR, interquartile range; LVEF, left ventricular ejection fraction; NYHA, New York Heart Association; SD, standard deviation; TIA, transient ischaemic attack; VT, ventricular tachycardia.

^a^Statistical comparison between Groups A and C.

*Table 2* summarizes the intra-procedural characteristics of all enrolled patients. Elective mechanical haemodynamic support was employed in 35 (28.5%) cases, and epicardial access was obtained in 14 (11.4%) patients. Patients in Group C were more likely to develop haemodynamically unstable VT/VF requiring rapid termination (via anti-tachycardia pacing or external cardioversion/defibrillation) compared with patients in Group A [*n* = 25 (75.8%) vs. *n* = 32 (41.6%), respectively; *P* = 0.002], but the frequency of acute haemodynamic decompensation necessitating premature termination of the procedure was comparable in all three groups [overall, *n* = 4 (3.3%)]. Similarly, there was no difference in the number of procedure-related complications in the three patient groups: *n* = 9 (7.3%) including three instances of acute pericardial tamponade, one pneumothorax resulting from chest compressions during an unstable rhythm, one instance of mixed shock requiring prolonged haemodynamic support, one instance of acute respiratory arrest requiring emergent veno-venous extracorporeal membrane oxygenation (VV ECMO) support, and three cases of Impella device–related complications (including axillary haematomas).

**Table 2:** Ablation procedure characteristics

Characteristic	Group A	Group B	Group C	All	*P*-value^a^
Mechanical support present, *n* (%)	19 (24.7)	5 (38.5)	11 (33.3)	35 (28.5)	0.360
ECMO present, *n* (%)	1 (1.3)	0 (0.0)	3 (9.1)	4 (3.3)	0.080
Impella present, *n* (%)	17 (22.1)	4 (30.8)	7 (21.2)	28 (22.8)	1.000
IABP present, *n* (%)	2 (2.6)	1 (7.7)	4 (12.1)	7 (5.7)	0.065
Pericardial access, *n* (%)	9 (11.7)	1 (7.7)	4 (12.1)	14 (11.4)	1.000
Number of VTs induced, median (IQR)	2 (1–3)	2 (2–3)	2 (1–3)	2 (1–3)	0.311
Number of VTs mapped, median (IQR)	1 (0–2)	1 (1–2)	2 (0–1)	1 (0–2)	0.082
Number of patients with at least one VT terminated with ablation, *n* (%)	34 (44.2)	6 (46.2)	8 (24.2)	48 (39.0)	0.056
Number of patients requiring external shock/ATP during the procedure, *n* (%)	32 (41.6)	7 (53.9)	25 (75.8)	64 (52.0)	0.002
Procedure time in minutes, mean ± SD	277.2 ± 100.1	255.7 ± 68.0	268.2 ± 76.1	272.5 ± 90.9	0.644

ATP, anti-tachycardia pacing; ECMO, extracorporeal membrane oxygenation; IABP, intra-aortic balloon pump; IQR, interquartile range; SD, standard deviation; VT, ventricular tachycardia.

^a^Statistical comparison between Groups A and C.

### Programmed ventricular stimulation

Primary recording system data were available for review for 62 patients (50.4%), while PVS assessment in 61 (50.0%) cases relied on procedural documentation. Post-ablation PVS was done in 93 (75.6%) cases. Up to 3 (or 4) extra-stimuli were employed in 69 (74.2%) procedures. Programmed ventricular stimulation was nearly always delivered from 1 pacing site (RV apex), while 2 or 3 pacing locations were utilized only in 12 (12.9%) and 2 (2.2%) cases, respectively. Additionally, ventricular burst pacing was used in 25 (26.9%) of procedures.

Most patients in Group A were non-inducible for any VT or VF [*n* = 71 (92.2%); of these, 53 patients received at least 3–4 extra-stimuli]. Conversely, rapid non-clinical VT [*n* = 4 (5.2%)] and VF [*n* = 2 (2.6%)] were infrequent in Group A. By definition, all patients in Group B were inducible for non-clinical VT with a tachycardia cycle length of >240 ms (*n* = 13). Finally, the vast majority of patients in Group C did not undergo any PVS testing [*n* = 30 (90.9%); of these, PVS was deferred because of existing or anticipated haemodynamic instability in 13 (43.3%) subjects, while the reasoning for PVS deferral was not stated in 17 (56.7%) patients], and only 3 (9.1%) participants had PVS repeated at the end of the case and remained inducible for clinical VT at the end of the procedure.

### Clinical outcomes

During a median follow-up period of 19 months (IQR 4–43 months), 82 (66.7%) patients reached the composite primary endpoint: 74 (60.2%) patients died, 4 (3.3%) obtained a durable LVAD, and 4 (3.3%) underwent heart transplant. The cause of death was directly related to the ablation procedure in two (2.7%) cases (cardiac tamponade). Arrhythmic or presumed arrhythmic deaths occurred in nine (12.2%) patients. Death from acute congestive heart failure occurred in 20 (27.0%) patients. The cause of death was non-cardiac or unknown in 22 (29.7%) and 21 (28.4%) patients, respectively. Ventricular tachycardia/VF recurrence resulting in appropriate ICD shocks occurred in 63 (51.2%) patients. A total of 12 patients (9.8%) were lost to follow-up [6 (7.8%) in Group A, 1 (7.7%) in Group B, and 5 (15.2%) in Group C].

**Figure 2:**
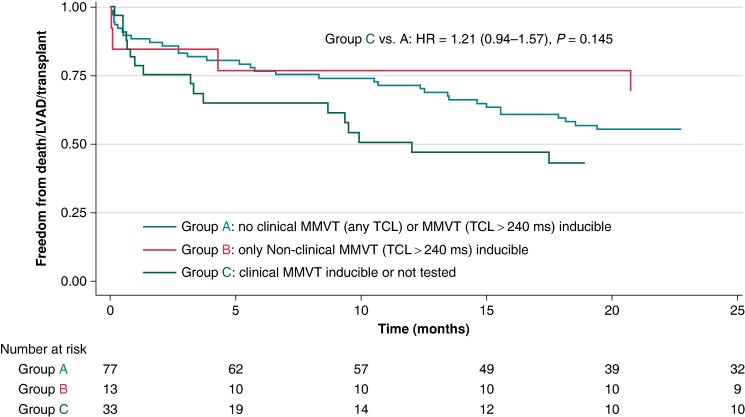
Kaplan–Meier analysis of time to death, LVAD implant, or transplant, stratified on acute post-procedural inducibility. HR, hazard ratio; LVAD, left ventricular assist device; MMVT, monomorphic ventricular tachycardia; TCL, tachycardia cycle length.

Patients in Group C had a comparable risk of death/LVAD/heart transplant as patients in Group A in unadjusted (HR 1.21, 95% CI 0.94–1.57, *P* = 0.145) and adjusted (HR 1.13, 95% CI 0.86–1.48, *P* = 0.388; adjusted for creatinine clearance, pericardial access, and presence of mechanical circulatory support) survival analyses (*Figure 2*). These findings were similar even when the analysis was restricted to 30-day outcomes (logistic regression): unadjusted odds ratio (OR) 1.43, 95% CI 0.83–2.46, *P* = 0.200; adjusted OR 1.17, 95% CI 0.62–2.23, *P* = 0.630.

**Figure 3:**
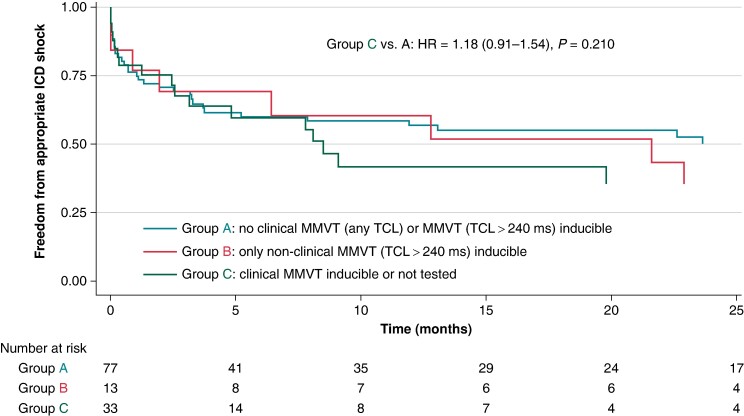
Kaplan-Meier analysis of time to appropriate ICD shock, stratified on acute post-procedural inducibility. HR, hazard ratio; ICD, implantable cardioverter-defibrillator; MMVT, monomorphic ventricular tachycardia; TCL, tachycardia cycle length.

Furthermore, there was no difference between Groups C and A with respect to the risk of appropriate ICD therapies or sustained VF/VF requiring external cardioversion/defibrillation (*Figure 3*) when analysed using a competing risk survival analysis model [unadjusted HR 1.18 (95% CI 0.91–1.54, *P* = 0.210) and adjusted HR 1.17 (95% CI 0.89–1.56, *P* = 0.262)] or when subjected to a 30-day landmark analysis [unadjusted OR 1.09 (95% CI 0.71–1.67, *P* = 0.400) and adjusted OR 1.00 (95% CI 0.64–1.57, *P* = 0.997)]. Additional 30-day landmark analyses showed no difference in secondary outcome rates between Groups A and B [unadjusted OR 0.93 (95% CI 0.46–1.89, *P* = 0.841) and adjusted OR 0.94 (95% CI 0.45–1.96, *P* = 0.862)]. Furthermore, when subjects were re-stratified into those inducible for any VT/VF vs. those completely non-inducible (excluding patients who were not tested), PVS was not a predictor of 30-day VT/VF recurrence [unadjusted OR 1.15 (95% CI 0.65–2.03, *P* = 0.628) and adjusted OR 1.07 (95% CI 0.60–1.93, *P* = 0.809)].

### Exploratory analysis

**Figure 4:**
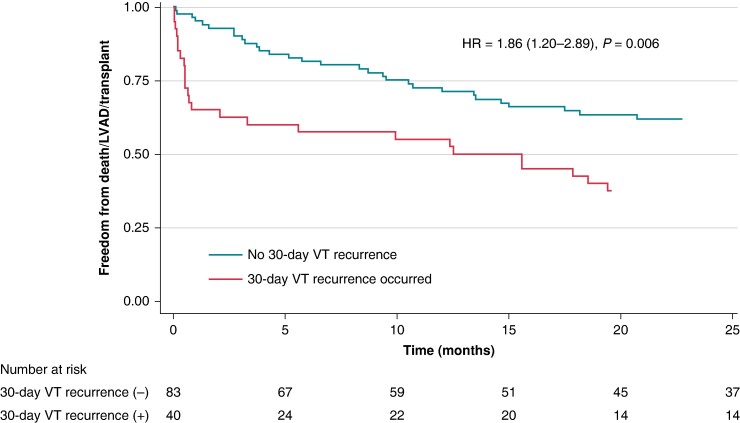
Kaplan–Meier analysis of time to death, LVAD implant, or transplant, stratified on the presence or absence of VT (or VF) recurrences (within 30 days post-ablation). HR, hazard ratio; LVAD, left ventricular assist device; VT, ventricular tachycardia.

Given the observed high mortality rate in the studied patient population and the apparent lack of correlation between acute VT inducibility and clinical outcomes, we wanted to see if another surrogate for ‘successful ablation’ may carry more clinical relevance. To this end, we explored the impact of early VT/VF recurrence on patients’ risk of death/LVAD/heart transplant. Indeed, the presence of at least one appropriate ICD (or external) shock in the first 30 days post-ablation was associated with a higher risk of the primary composite endpoint in unadjusted (HR 1.86, 95% CI 1.20–2.89, *P* = 0.006) and adjusted (HR 1.86, 95% CI 1.19–2.91, *P* = 0.006) analyses (*Figure 4*). Of note, PVS results also did not predict 30-day recurrent VT/VF in unadjusted (OR 1.09, 95% CI 0.71–1.67, *P* = 0.692) and adjusted (OR 1.00, 95% CI 0.64–1.57, *P* = 0.997) analyses.

## Discussion

### Programmed ventricular stimulation in high-risk patients

This analysis demonstrated VT non-inducibility by PVS following VT ablation remains an imperfect procedural endpoint for predicting VT/VF recurrence or a mortality equivalent in complex high-risk patients (PAINESD > 17). However, early recurrences of VT/VF were associated with high short-term mortality.

Non-inducibility by PVS has long been used as a stipulated procedural endpoint in many VT ablation trials although actual post-ablation non-inducibility is rarely achieved in all trial subjects. Furthermore, even if non-inducibility is indeed achieved in most patients, the high rates of VT recurrence reported in some of the landmark trials (ranging from 12% to as high as 53%) attest to the imperfect nature of PVS as a predictor of clinical outcomes.^5–7,9,16,17^

That said, more granular information about the predictive value of PVS can be gleaned from comprehensive sub-analyses of large multicentre registries. For example, a multivariate analysis of the 2061 patients enrolled in the International VT Ablation Center Collaborative Group study (IVTCC)^8^ revealed a strong association between acute post-ablation non-inducibility and lower mortality (HR 1.97, 95% CI 1.25–3.12, *P* = 0.004) along with lower VT recurrence rates (HR 1.58, 95% CI 1.08–2.32, *P* = 0.019). However, compared with patients analysed in this manuscript, the IVTCC cohort appeared to be less medically complex [for example, less NYHA Class III/IV heart failure (31.4% vs. 78.9%), less frequent ICM (53.1% vs. 92.7%), and less frequent VT storm (33.2% vs. 83.7%)]. Similar observations were made in a number of other cohorts, all of which generally enrolled less moribund subjects compared with our study.^18–21^ Conversely, a sub-analysis of the more recently published BERLIN-VT trial failed to show significant association between acute VT inducibility and clinical outcomes.^13^

A 2013 study by Della Bella et al. was one of the first large-scale endeavours specifically designed to validate the utility of acute PVS inducibility after VT ablation.^12^ This was a large prospective cohort study that stratified 482 subjects into 3 groups based on post-procedural inducibility (clinical VT inducible vs. clinical VT not inducible but non-clinical VT inducible vs. all VT not inducible). Clinical VT inducibility was associated with both cardiac mortality (HR 2.099, *P* = 0.04) and VT recurrence (HR 4.030, *P* < 0.001). Once again, the studied cohort was less medically complex than the patients analysed in our retrospective work [lower mean age (62.1 ± 14.0 vs. 69.9 ± 6.9 years), less NYHA Class III/IV heart failure (28.6% vs. 78.9%), less frequent ICM (54.9% vs. 92.7%), less frequent VT storm (28.6% vs. 83.7%), and less frequent end-stage renal disease (ESRD; 0.01% vs. 4.1%)]. Interestingly, in a sub-analysis of high-risk patients, acute clinical VT inducibility became an even more potent predictor of cardiac mortality and VT recurrence. Such diverging findings may be explained by differences in study design: for example, the Della Bella study relied on a different risk stratification algorithm (reflecting haemodynamics, VT burden, and comorbidities), excluded patients in whom PVS was not performed, and exhibited a high acute success rate (77% of patients were non-inducible for any VT). The findings of the Della Bella study were partially replicated by a 2015 multicentre study of 1064 patients with ICM.^22^ In this work, acute non-inducibility of any VT was associated with improved mortality (HR 0.65, 95% CI 0.53–0.79, *P* < 0.001). Interestingly, this effect may have been driven by early mortality (<3 months post-ablation)—a phenomenon that is qualitatively similar to the mortality effect of early VT recurrence documented in our study (*Figure 3*).

Post-ablation VT inducibility is a stochastic phenomenon, so it is not surprising that PVS may not be a perfect prognosticator of clinical outcomes. Furthermore, PVS methodology (e.g. number of pacing sites and extra-stimuli used) is not uniform in the *de facto* clinical practice across different EP labs and individual operators or in the design of various prospective study protocols; this inherently introduces additional variability into retrospective and prospective studies, respectively. Even so, as discussed, the bulk of clinical evidence supports the utility of post-ablation PVS in most patients, many of whom have a relatively low burden of comorbidities. In contrast, our present analysis exclusively pertained to high-risk individuals, in whom PVS outcomes appear to carry little prognostic value. These patients frequently suffered from multiple competing mortality risk factors that may ‘drown out’ the prognostic utility of post-ablation stimulation. It is less clear why PVS outcomes were not predictive of VT/VF recurrences in this patient cohort, although several explanations can be postulated. One possibility is that the mechanism of VT/VF recurrences may be different in patients with advanced cardiac disease (e.g. triggered activity in the setting of advanced heart failure).^23^ Furthermore, PVS outcomes may be highly dependent on specific cardiac substrate. For example, in a sub-study of the VANISH trial, patients with inferior and non-inferior infarcts had comparable responses to end-procedural PVS, yet clinical benefit from ablation (compared with escalation of anti-arrhythmic drug therapy) was only seen in the latter group.^24^ Thus, the patients enrolled in our study may be enriched for cardiac substrate for which PVS results are less interpretable. Alternatively, the lack of association between PVS and VT/VF recurrences may have been an issue of statistical power: the rate of the competing endpoint (mortality equivalent) may simply have been too high in this patient cohort to allow for a meaningful analysis of VT/VF recurrence rates. Future studies are needed to identify more accurate procedural endpoints for this subset of patients (including post-ablation non-invasive programmed stimulation). The high mortality in the studied cohort also highlights the importance of appropriate patient selection and early evaluation for advanced heart failure management in high-risk individuals, as outlined in the recently published consensus statement.^25^

### Other predictors of long-term outcomes

Our exploratory analysis identified VT/VF recurrence defined as appropriate ICD shock (or VT/VF requiring external cardioversion/defibrillation) within 30 days of ablation as a predictor of the combined primary endpoint of mortality, durable LVAD, or cardiac transplant. Unsurprisingly, this result is consistent with the existing literature. For example, a similar observation was already made in the Thermocool VT trial, where post-ablation VT recurrences correlated with subsequent mortality (HR 3.07, 95% CI 1.23–7.75, *P* = 0.016).^26^ Similarly, the IVTCC registry confirmed a strong relationship between VT recurrence and transplant-free survival (HR 6.90, 95% CI 5.28–9.02, *P* < 0.001).^8^ Interestingly, the effect size of this association was largest in patients with reduced LVEF (<30%). Importantly, the relationship between VT recurrence and death is not necessarily causal; in fact, the vast majority of deaths in our study were not arrhythmia related.

### Study limitations

The large majority of Group C patients had no PVS repeated at the end of the procedure. Since the lack of end-procedural PVS likely reflects a perception of clinical futility by the operator, we chose to include these patients in our analysis as part of Group C. This decision is also supported by a prior large analysis that demonstrated individuals not tested for VT inducibility via PVS had higher VT recurrence rates compared with those with documented non-inducibility (HR 2.08, 95% CI 1.19–3.64, *P* = 0.006).^21^ In addition, the small size of Group B limited our ability to make conclusions regarding predictive capacity of patients in which clinical VT was not inducible but non-clinical VTs remained inducible. The possible issue of insufficient statistical power may also extend to Groups A and C; *Figure 2* shows an early trend towards higher event rates in Group C, which may have reached statistical significance had more subjects been available for analysis. It also needs to be stated that the predictive capacity of PVS can be exquisitely sensitive to the exact PVS protocol used (especially the final coupling intervals reached);^27^ consequently, the non-uniform nature of PVS protocols used in this study may adversely impact the validity of our findings. Furthermore, the employed ablation strategy (VT induction and mapping whenever possible, followed by substrate modification targeting abnormal potentials and sites with favourable pace map morphologies) may have impacted our study. Some substrate-based ablation techniques carry their own intrinsic endpoints (e.g. ablation of decrement-evoked potentials) that can lead to excellent clinical outcomes.^28,29^ However, these techniques were not uniformly employed in our study. Therefore, whether the findings from this study can be applicable to other ablation approaches such as pure substrate-based ablation with no attempt at VT induction warrants further investigation. Additionally, we acknowledge that there were uneven rates of loss to follow-up in the three studied cohorts, which may bias our observations. Finally, it is important to state that this analysis only pertains to narrowly defined high-risk patients (i.e. those with PAINESD score > 17) and our observations may not apply to other patient strata.

### Conclusions

The results of acute post-ablation PVS did not correlate with long-term outcomes of VT recurrence, mortality, or mortality equivalent, in high-risk patients (PAINESD > 17). Although prior literature provides evidence for the predictive capacity of PVS inducibility, such capacity appears to diminish in patients with multiple advanced comorbidities.

Further research is needed to better study and develop alternate procedural endpoints for VT ablation. Our study also highlights the overall high rate of unfavourable outcomes in this high-risk population and, consequently, the importance of careful patient selection when identifying VT ablation candidates.

## Data Availability

The data underlying this article will be shared on reasonable request to the corresponding author.

## References

[1] Cronin EM, Bogun FM, Maury P, Peichl P, Chen M, Namboodiri N et al 2019 HRS/EHRA/APHRS/LAHRS expert consensus statement on catheter ablation of ventricular arrhythmias: executive summary. J Arrhythm 2020;36:1–58. 32071620 10.1002/joa3.12264 PMC7011820

[2] Natale A, Zeppenfeld K, Della Bella P, Liu X, Sabbag A, Santangeli P et al Twenty-five years of catheter ablation of ventricular tachycardia: a look back and a look forward. Europace 2023;25:euad225. 37622589 10.1093/europace/euad225 PMC10451002

[3] Wellens HJ, Schuilenburg RM, Durrer D. Electrical stimulation of the heart in patients with ventricular tachycardia. Circulation 1972;46:216–26. 4114692 10.1161/01.cir.46.2.216

[4] Sultan A, Futyma P, Metzner A, Anic A, Richter S, Roten L et al Management of ventricular tachycardias: insights on centre settings, procedural workflow, endpoints, and implementation of guidelines—results from an EHRA survey. Europace 2024;26:euae030. 38363995 10.1093/europace/euae030 PMC10872712

[5] Sapp JL, Wells GA, Parkash R, Stevenson WG, Blier L, Sarrazin JF et al Ventricular tachycardia ablation versus escalation of antiarrhythmic drugs. N Engl J Med 2016;375:111–21. 27149033 10.1056/NEJMoa1513614

[6] Reddy VY, Reynolds MR, Neuzil P, Richardson AW, Taborsky M, Jongnarangsin K et al Prophylactic catheter ablation for the prevention of defibrillator therapy. N Engl J Med 2007;357:2657–65. 18160685 10.1056/NEJMoa065457 PMC2390777

[7] Tung R, Josephson ME, Reddy V, Reynolds MR, Investigators S-V. Influence of clinical and procedural predictors on ventricular tachycardia ablation outcomes: an analysis from the substrate mapping and ablation in Sinus Rhythm to Halt Ventricular Tachycardia Trial (SMASH-VT). J Cardiovasc Electrophysiol 2010;21:799–803. 20132389 10.1111/j.1540-8167.2009.01705.x

[8] Tung R, Vaseghi M, Frankel DS, Vergara P, Di Biase L, Nagashima K et al Freedom from recurrent ventricular tachycardia after catheter ablation is associated with improved survival in patients with structural heart disease: an International VT Ablation Center Collaborative Group study. Heart Rhythm 2015;12:1997–2007. 26031376 10.1016/j.hrthm.2015.05.036 PMC4549209

[9] Calkins H, Epstein A, Packer D, Arria AM, Hummel J, Gilligan DM et al Catheter ablation of ventricular tachycardia in patients with structural heart disease using cooled radiofrequency energy: results of a prospective multicenter study. J Am Coll Cardiol 2000;35:1905–14. 10841242 10.1016/s0735-1097(00)00615-x

[10] Carbucicchio C, Santamaria M, Trevisi N, Maccabelli G, Giraldi F, Fassini G et al Catheter ablation for the treatment of electrical storm in patients with implantable cardioverter-defibrillators: short- and long-term outcomes in a prospective single-center study. Circulation 2008;117:462–9. 18172038 10.1161/CIRCULATIONAHA.106.686534

[11] Della Bella P, Riva S, Fassini G, Giraldi F, Berti M, Klersy C et al Incidence and significance of pleomorphism in patients with postmyocardial infarction ventricular tachycardia. Acute and long-term outcome of radiofrequency catheter ablation. Eur Heart J 2004;25:1127–38. 15231371 10.1016/j.ehj.2004.01.021

[12] Della Bella P, Baratto F, Tsiachris D, Trevisi N, Vergara P, Bisceglia C et al Management of ventricular tachycardia in the setting of a dedicated unit for the treatment of complex ventricular arrhythmias: long-term outcome after ablation. Circulation 2013;127:1359–68. 23439513 10.1161/CIRCULATIONAHA.112.000872

[13] Willems S, Tilz RR, Steven D, Kaab S, Wegscheider K, Geller L et al Preventive or deferred ablation of ventricular tachycardia in patients with ischemic cardiomyopathy and implantable defibrillator (BERLIN VT): a multicenter randomized trial. Circulation 2020;141:1057–67. 32000514 10.1161/CIRCULATIONAHA.119.043400

[14] Santangeli P, Frankel DS, Tung R, Vaseghi M, Sauer WH, Tzou WS et al Early mortality after catheter ablation of ventricular tachycardia in patients with structural heart disease. J Am Coll Cardiol 2017;69:2105–15. 28449770 10.1016/j.jacc.2017.02.044

[15] Sosa E, Scanavacca M, d'Avila A, Pilleggi F. A new technique to perform epicardial mapping in the electrophysiology laboratory. J Cardiovasc Electrophysiol 1996;7:531–6. 8743758 10.1111/j.1540-8167.1996.tb00559.x

[16] Kuck KH, Schaumann A, Eckardt L, Willems S, Ventura R, Delacretaz E et al Catheter ablation of stable ventricular tachycardia before defibrillator implantation in patients with coronary heart disease (VTACH): a multicentre randomised controlled trial. Lancet 2010;375:31–40. 20109864 10.1016/S0140-6736(09)61755-4

[17] Essebag V, Joza J, Nery PB, Doucette S, Nault I, Rivard L et al Prognostic value of noninducibility on outcomes of ventricular tachycardia ablation: a VANISH substudy. JACC Clin Electrophysiol 2018;4:911–9. 30025692 10.1016/j.jacep.2018.03.013

[18] Sauer WH, Zado E, Gerstenfeld EP, Marchlinski FE, Callans DJ. Incidence and predictors of mortality following ablation of ventricular tachycardia in patients with an implantable cardioverter-defibrillator. Heart Rhythm 2010;7:9–14. 19939743 10.1016/j.hrthm.2009.09.014

[19] Dinov B, Fiedler L, Schonbauer R, Bollmann A, Rolf S, Piorkowski C et al Outcomes in catheter ablation of ventricular tachycardia in dilated nonischemic cardiomyopathy compared with ischemic cardiomyopathy: results from the Prospective Heart Centre of Leipzig VT (HELP-VT) study. Circulation 2014;129:728–36. 24211823 10.1161/CIRCULATIONAHA.113.003063

[20] Kumar S, Romero J, Mehta NK, Fujii A, Kapur S, Baldinger SH et al Long-term outcomes after catheter ablation of ventricular tachycardia in patients with and without structural heart disease. Heart Rhythm 2016;13:1957–63. 27392945 10.1016/j.hrthm.2016.07.001

[21] Siontis KC, Kim HM, Stevenson WG, Fujii A, Bella PD, Vergara P et al Prognostic impact of the timing of recurrence of infarct-related ventricular tachycardia after catheter ablation. Circ Arrhythm Electrophysiol 2016;9:e004432. 27923805 10.1161/CIRCEP.116.004432 PMC5823603

[22] Yokokawa M, Kim HM, Baser K, Stevenson W, Nagashima K, Della Bella P et al Predictive value of programmed ventricular stimulation after catheter ablation of post-infarction ventricular tachycardia. J Am Coll Cardiol 2015;65:1954–9. 25913000 10.1016/j.jacc.2015.02.058

[23] Wang Z, Taylor LK, Denney WD, Hansen DE. Initiation of ventricular extrasystoles by myocardial stretch in chronically dilated and failing canine left ventricle. Circulation 1994;90:2022–31. 7522991 10.1161/01.cir.90.4.2022

[24] Samuel M, Rivard L, Nault I, Gula L, Essebag V, Parkash R et al Comparative effectiveness of ventricular tachycardia ablation vs. escalated antiarrhythmic drug therapy by location of myocardial infarction: a sub-study of the VANISH trial. Europace 2022;24:948–58. 34964475 10.1093/europace/euab298 PMC9282915

[25] Lenarczyk R, Zeppenfeld K, Tfelt-Hansen J, Heinzel FR, Deneke T, Ene E et al Management of patients with an electrical storm or clustered ventricular arrhythmias: a clinical consensus statement of the European Heart Rhythm Association of the ESC-endorsed by the Asia-Pacific Heart Rhythm Society, Heart Rhythm Society, and Latin-American Heart Rhythm Society. Europace 2024;26:euae049. 38584423 10.1093/europace/euae049 PMC10999775

[26] Stevenson WG, Wilber DJ, Natale A, Jackman WM, Marchlinski FE, Talbert T et al Irrigated radiofrequency catheter ablation guided by electroanatomic mapping for recurrent ventricular tachycardia after myocardial infarction: the multicenter Thermocool ventricular tachycardia ablation trial. Circulation 2008;118:2773–82. 19064682 10.1161/CIRCULATIONAHA.108.788604

[27] Kovoor JG, Deshmukh T, von Huben A, Marschner SL, Byth K, Chow CK et al Optimizing electrophysiology studies to prevent sudden cardiac death after myocardial infarction. Europace 2023;25:euad219. 37470454 10.1093/europace/euad219 PMC10374980

[28] Porta-Sánchez A, Jackson N, Lukac P, Kristiansen SB, Nielsen JM, Gizurarson S et al Multicenter study of ischemic ventricular tachycardia ablation with decrement-evoked potential (DEEP) mapping with extra stimulus. JACC Clin Electrophysiol 2018;4:307–15. 30089555 10.1016/j.jacep.2017.12.005

[29] Al-Sheikhli J, Winter J, Luque IR, Lambiase PD, Orini M, Porta-Sanchez A et al Optimization of decrementing evoked potential mapping for functional substrate identification in ischaemic ventricular tachycardia ablation. Europace 2023;25:euad092. 37032650 10.1093/europace/euad092 PMC10228600

